# Inflammatory Responses Are Not Sufficient to Cause Delayed Neuronal Death in ATP-Induced Acute Brain Injury

**DOI:** 10.1371/journal.pone.0013756

**Published:** 2010-10-29

**Authors:** Hey-Kyeong Jeong, Kyung-min Ji, Beomsue Kim, Jun Kim, Ilo Jou, Eun-hye Joe

**Affiliations:** 1 Neuroscience Graduate Program, Ajou University School of Medicine, Suwon, Korea; 2 Department of Pharmacology, Ajou University School of Medicine, Suwon, Korea; 3 Brain Disease Research Center, Ajou University School of Medicine, Suwon, Korea; 4 Chronic Inflammatory Disease Research Center, Ajou University School of Medicine, Suwon, Korea; Brigham and Women's Hospital, Harvard Medical School, United States of America

## Abstract

**Background:**

Brain inflammation is accompanied by brain injury. However, it is controversial whether inflammatory responses are harmful or beneficial to neurons. Because many studies have been performed using cultured microglia and neurons, it has not been possible to assess the influence of multiple cell types and diverse factors that dynamically and continuously change in vivo. Furthermore, behavior of microglia and other inflammatory cells could have been overlooked since most studies have focused on neuronal death. Therefore, it is essential to analyze the precise roles of microglia and brain inflammation in the injured brain, and determine their contribution to neuronal damage in vivo from the onset of injury.

**Methods and Findings:**

Acute neuronal damage was induced by stereotaxic injection of ATP into the substantia nigra pars compacta (SNpc) and the cortex of the rat brain. Inflammatory responses and their effects on neuronal damage were investigated by immunohistochemistry, electron microscopy, quantitative RT-PCR, and stereological counting, etc. ATP acutely caused death of microglia as well as neurons in a similar area within 3 h. We defined as the core region the area where both TH^+^ and Iba-1^+^ cells acutely died, and as the penumbra the area surrounding the core where Iba-1^+^ cells showed activated morphology. In the penumbra region, morphologically activated microglia arranged around the injury sites. Monocytes filled the damaged core after neurons and microglia died. Interestingly, neither activated microglia nor monocytes expressed iNOS, a major neurotoxic inflammatory mediator. Monocytes rather expressed CD68, a marker of phagocytic activity. Importantly, the total number of dopaminergic neurons in the SNpc at 3 h (∼80% of that in the contralateral side) did not decrease further at 7 d. Similarly, in the cortex, ATP-induced neuron-damage area detected at 3 h did not increase for up to 7 d.

**Conclusions:**

Different cellular components (microglia, astrocytes, monocytes, and neutrophils) and different factors (proinflammatory and neurotrophic) could be produced in inflammatory processes depending on the nature of the injury. The results in this study suggest that the inflammatory responses of microglia and monocytes in response to ATP-induced acute injury could not be neurotoxic.

## Introduction

Brain inflammation is emerging as an important target of drug development in the treatment of acute and degenerative brain diseases. Several studies have shown that brain inflammation accompanying injury is mainly mediated by resident microglia, and inflammatory responses are harmful to surrounding neurons due to the cytotoxicity of mediators, such as nitric oxide (NO) and TNF-α [1]–[8]. However, other studies have challenged this general concept. Firstly, brain inflammation may be a complex process involving blood inflammatory cells as well as microglia. We and others have reported that neutrophils and/or monocytes infiltrate traumatic, ischemic, and LPS-injected brain and that neutrophils rather than microglia express neurotoxic iNOS and TNF-α [9]–[19]. In animal models of Alzheimer's disease, monocytes infiltrate brains [20], [21]. Secondly, neuroprotective roles of inflammatory responses are reported. Microglia/monocytes phagocyte dying cells and debris, and produce neurotrophic factors, such as transforming growth factor-β1 (TGF-β1), neutrophin-3 (NT-3), and brain-derived neurotrophic factor (BDNF) [22]–[26]. Because most studies have been performed using cultured microglia and neurons, it has not been possible to assess the influence of multiple cell types and diverse factors that dynamically and continuously change in vivo. Furthermore, most such studies have focused on neuronal death; thus, the behavior of microglia and other inflammatory cells could have been overlooked. Therefore, it is essential to analyze the precise roles of microglia and brain inflammation in the injured brain, and determine their contribution to neuronal damage in vivo from the onset of injury.

ATP has been considered an endogenous factor that induces neuronal damage and inflammatory responses. Under some pathological conditions, such as ischemia and hypoxia, the extracellular ATP level rises due to release from damaged cells [27]–[30]. ATP directly induces neuronal death [31]. ATP also activates microglia: microglia rapidly move their processes towards ATP [32], and produce IL-1β, TNF-α, and ROS in response to ATP [33]–[36]. Furthermore, blocking of ATP receptors reduces brain injury [27]–[29], [37]. Thus, ATP is a suitable insult to mimic in vivo brain injury.

In this study, we injected ATP into the substantia nigra pars compacta (SNpc, Parkinson's disease- related brain area) and the cortex, and investigated inflammatory processes and their effects on neuronal damage. Interestingly, microglia died as early as neurons within the core region, indicating that microglia are as vulnerable to insult as neurons. Microglia in the penumbra region surrounded the injury sites, and cells highly expressing CD45^+^ (a marker of monocytes) appeared at around 2 d. However, neuron-damage area detected at 3 h did not increase further for up to 7 d in both SNpc and cortex. Therefore, microglia/monocytes appear to isolate injury sites and/or remove damaged cells rather than causing delayed neuronal death at least in the ATP-injected brain.

## Materials and Methods

### Ethics Statement

All experiments were performed in accordance with the approved animal protocols and guidelines established by the Ajou University School of Medicine Ethics Review Committee for animal experiments, and all animal work was approved by the Ethical Committee for Animal Research of Ajou University (Amc-28).

### Stereotaxic surgery and drug injection

SD rats were anesthetized by injection of chloral hydrate (0.4 mg/kg, i.p.), and positioned in stereotaxic apparatus (David Kopf Instruments, Tujunga, CA). ATP (10–1000 nmol in 2 µl sterile PBS; Sigma, St. Louis, MO) was unilaterally administered into the right SNpc (AP, −5.3 mm; ML, −2.3 mm; DV, −7.6 mm from bregma) and cortex (AP, +0.7 mm; ML, −2.0 mm; DV, −2.0 mm from bregma), according to the atlas of Paxinos and Watson [38]. All animals were injected using a Hamilton syringe equipped with a 30-gauge blunt needle to minimize mechanical damage attached to a syringe pump (KD Scientific, New Hope, PA). ATP was infused at a rate of 0.4 µl/min. After injection, the needle was held in place for an additional 5 min before removal. PBS-injected animals or contralateral sides were used as control.

### Tissue preparation

Rats were anesthetized and transcardially perfused with saline solution containing 0.5% sodium nitrate and heparin (10 U/ml), followed by 4% paraformaldehyde in 0.1 M phosphate buffer, pH 7.2, for tissue fixation. Brains were obtained and post-fixed overnight at 4°C in 4% paraformaldehyde. Fixed brains were stored at 4°C in 30% sucrose solution until they sank. Six separate series of 30 µm coronal brain sections (50 µm for stereological counting) were obtained with a sliding microtome (Microm, Walldorf, Germany). For RNA preparation, rats were anesthetized and transcardially perfused with saline solution without prarformaldehyde. Brains were obtained and sliced with Rat Brain Slicer Matrix (1.0 mm slice intervals, RBM-4000C, ASI Instruments, Warren, MI) and a razor blade. The slice including the needle injection spot was selected, and tissue blocks (2×2×2 mm^3^) just below the needle tip were collected, and stored at −70°C until use.

### Monocytes isolation and transplantation

Rat blood monocytes were isolated by density gradient centrifugation [39] using endotoxin-free Ficoll-Paque PLUS (GE Healthcare, Uppsala, Sweden). Briefly, blood was mixed with 2.5% dextran in PBS (blood: dextran solution, 1∶4) for 1 h at RT. The plasma layer was loaded onto Ficoll, and centrifuged at 400× g for 30 min. The monocytes layer displaying a white band at the interface was carefully collected and washed 3 times with PBS. Then, monocytes were stained with 10 µM CFDA-SE for 10 min at 37°C. Cells were washed 3 times with PBS, resuspended in PBS. CFDA-labeled monocytes (1×10^8^ cells/300 µl) were transplanted through the tail vein at 12 h after ATP injection into the SNpc. Rats were sacrificed 2 d after ATP injection.

### Immunohistochemistry

For 3, 3′-diaminobenzidine (DAB) staining, serial sections were rinsed three times with PBS, treated with 3% H_2_O_2_ for 5 min, and rinsed with PBS containing 0.2% Triton X-100 (PBST). Non-specific binding was blocked with 1% BSA in PBST. Sections were incubated overnight at room temperature with primary antibodies against tyrosine hydroxylase (TH; 1∶2000 dilution; Pelfreeze Biologicals, Rogers, AR), ionized calcium binding adaptor molecule 1 (Iba-1; 1∶1000; Wako Pure Chemical Industries, Osaka, Japan), myeloperoxidase (MPO; 1∶1000; Dako, Glostrup, Denmark), CD45 (1∶200; AbD Serotec, Oxford, UK), CD11b (OX-42; 1∶200; Serotec, Oxford, UK), Ki-67 (1∶100; Abcam, Cambridge, UK), interleukin-1β (IL-1β; 1∶200; R&D systems, MN), inducible nitric oxide synthase (iNOS; 1∶200; Abcam, Cambridge, UK), CD68 (1∶200; AbD Serotec, oxford, UK) or Neuronal Nuclei (NeuN; 1: 300; Chemicon, CA, USA). Following rinsing in PBST, sections were incubated with biotinylated secondary antibodies (Vector Laboratories, Burlingame, CA) for 1 h and the avidin/biotin system (Vector Laboratories, Burlingame, CA) for 1 h and visualized using DAB solution (0.05% DAB and 0.003% hydrogen peroxide in 0.1 M PB). For double-labeling with TH and Iba-1, sections were stained with TH antibody following the above procedure, and visualized with DAB (brown product). And then, same sections were washed in PBS, blocked with 1% BSA, incubated with Iba-1 antibody, and visualized using DAB/nickel sulfate solution (dark purple products) according to the manufacturer's guidance. Next, sections were mounted on gelatin-coated slides, and examined under a bright field microscope (Olympus Optical, BX51, Tokyo, Japan). Bright field images were obtained using PictureFrame Application 2.3 software. For immunofluorescence staining, sections were washed twice in PBS, treated with 1% BSA, and incubated with combinations of antibodies against Iba-1, iNOS, interleukin-1β, CD68, CD11b, CD45, Ki67, TH and NeuN. Visualization was performed with Alexa Fluor488- or Alexa Fluor555- conjugated secondary antibodies (1∶600 dilution; Invitrogen, Eugene, OR, USA). DAPI (Vector Laboratories, Burlingame, CA) was used to detect nuclei. Sections were analyzed under a confocal microscope (Carl Zeiss, Germany) with 40× water and 63× oil immersion objectives at 20°C, and images were captured using Confocal software (LSM Image Browser).

### Electron Microscopy

At 3 h after ATP injection, brains were obtained and sliced (1.0 mm slice) with Rat Brain Slicer Matrix and a razor. ATP-injected SNpc tissue blocks (smaller than 1×1×1 mm^3^) were collected and post-fixed overnight at 4°C using Karnovsky's fixative solution (2% paraformaldehyde, 2% glutaraldehyde, 0.5% calcium chloride in cacodylate buffer, pH 7.2), washed with cacodylate buffer, dehydrated in a series of graded ethanol washes, and embedded in Epon mixture. Semi-thin (1 µm thickness) sections were cut and stained with toluidine blue for examination under a light microscope to determine tissue orientation. Ultrathin sections (70∼80 nm) were obtained using a Reichert Jung Ultracut S microtome (Leica, Vienna, Austria), mounted on grids, stained with uranyl acetate and lead citrate, and analyzed under a Zeiss EM 902 A electron microscope (Leo, Oberkohen, Germany).

### Quantitative real-time PCR (Q-PCR)

Total RNA was isolated using an easy-BLUE RNA Extraction Kit (iNtRON, Sungnam, Korea), and cDNA was prepared using Reverse Transcription Master Premix (ELPisbio, Taejeon, Korea), according to the manufacturers' instructions. For Q-PCR, approximately 50 ng cDNA was analyzed using a KAPA SYBR FAST qPCR Kit (KAPA Biosystems, Boston, MA) and a Corbett Rotor-Gene 6000 real-time rotary analyzer (Corbett Research, Mortlake, NSW, Australia). Specific primers for IL-1β, TNF-α, IL-6, and glyceraldehyde-3-phosphate dehydrogenase (GAPDH) used in Q-PCR are shown in Table 1. Q-PCR conditions were as follows: 95°C for 30 s, followed by 40 cycles of 95°C for 3 s (denaturation), 55°C for 20 s (annealing), and 72°C for 3 s (elongation). For TNF-α, the annealing conditions were modified to include a 63°C–59°C touch-down protocol, decreasing temperature by 0.5°C per cycle for the first 8 cycles. That a single product was amplified under the conditions used was confirmed by performing a melting curve analysis for each primer pair, using a melt ramp of 72°C–95°C and raising the temperature by 1°C at each step (5 s/step). Amplified products were also verified by electrophoresis on 1.5% agarose gels with GelRed (Biotium, USA) staining. Results were normalized to GAPDH, used as a reference.

**Table 1:** List of primer sequences for Q-PCR.

Gene	Sequence (5′-3′)
IL-1β	**F-** TGATGTTCCCATTAGACAGC
	**R-** GAGGTGCTGATGTACCAGTT
TNF-α	**F-** GTAGCCCACGTCGTAGCAAA
	**R-** CCCTTCTCCAGCTGGGAGAC
IL-6	**F-** AAAATCTGCTCTGGTCTTCTGG
	**R-** GGTTTGCCGAGTAGACCTCA
GAPDH	**F-** TCCCTCAAGATTGTCAGCAA
	**R-** AGATCCACAACGGATACATT

F, Forward primer; R, Reverse primer.

### Stereological analysis of dopaminergic neurons

Coronal brain sections (50 µm) were obtained and stained with TH antibodies. The number of TH^+^ neurons in the entire SNpc was counted under a bright field microscope (Olympus Optical, BX51, Tokyo, Japan) using Stereo Investigator software (MBF Bioscience, Williston, VT). Counting frames (100 µm ×100 µm) were randomly placed on the SNpc, and TH^+^ neurons counted under a 40x objective.

### Statistical Analysis

Statistical data were expressed as means ± SEM. The significance of data was analyzed with one-way ANOVA using the Statistical Package for Social Sciences (SPSS) 12.0.

## Results

### Time-dependent behavior of inflammatory cells in response to ATP injection

**Figure 1:**
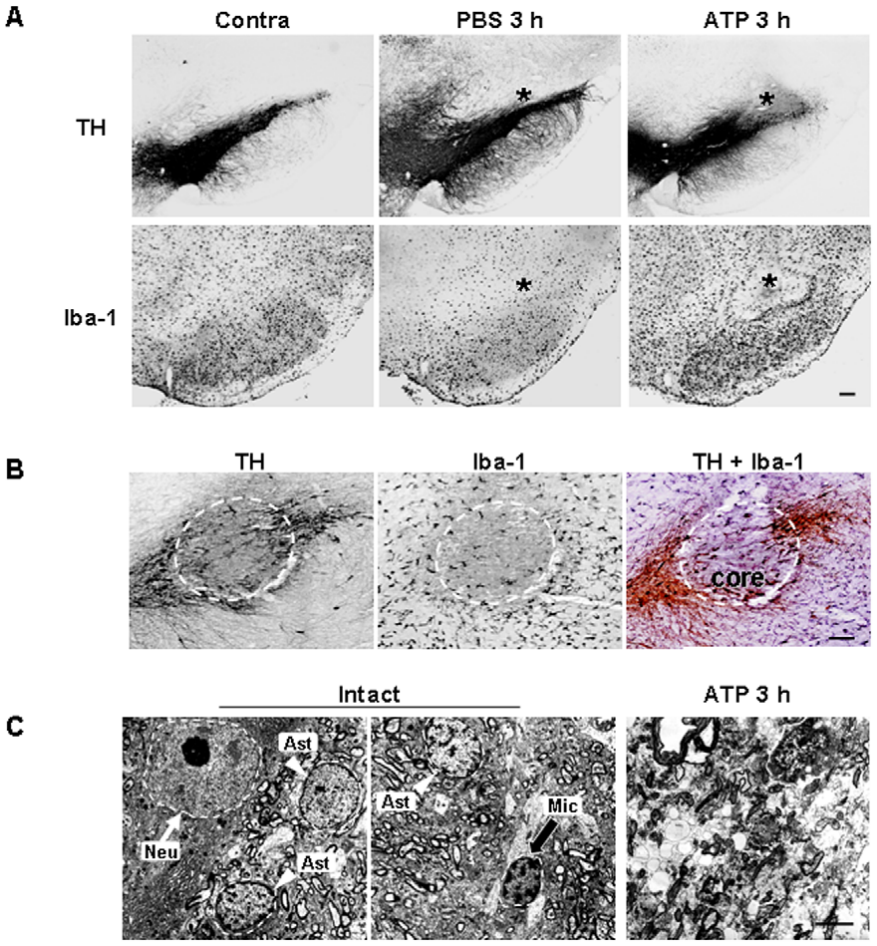
Death of dopaminergic neurons and microglia in the SNpc induced by ATP. (**A**) ATP (100 nmol in 2 µl PBS) or PBS (2 µl) was unilaterally injected into SNpc (*, injection sites), and brains were obtained after 3 h. Brain sections (30 µm thickness) of the midbrain including the entire SN were prepared, every sixth serial section selected and stained with TH (upper panel) and Iba-1 (lower panel) antibodies, and visualized with biotin-conjugated secondary antibodies and enzymatic detection with the avidin/biotin system unless indicated. At 100 nmol, mild neuronal and microglial damage occurred, thus, 100 nmol ATP was employed for *in vivo* experiments in this study. Photographs of the most damaged sections were obtained. The contralateral side (contra) and PBS-injected rat brain sections were used as control. (**B**) Serial sections obtained at 3 h were labeled with TH (left panel), Iba-1 (middle panel), and TH/Iba-1 (right panel) antibodies. For visualization of the double-labeling, color reactions using DAB (for TH) and DAB/nickel sulfate (for Iba-1) were applied. Dotted lines indicated damage areas. (**C**) Brain tissue obtained 3 h post ATP treatment was subjected to electron microscopy, as described in “Materials and Methods”. Nuclei of neuron (N, white arrow), astrocytes (A, white arrowhead), and microglia (M, black arrow) were shown in intact rat brain whereas cellular structures were severely disrupted in ATP-injected brain. Data in this study are representative of at least 5 animals. Scale bars, 200 µm (A); 100 µm (B); 5 µm (C).

To evaluate brain inflammatory cell behavior in response to injury, we injected ATP into the substantia nigra pars compacta (SNpc), the brain area closely related to Parkinson's disease. ATP was selected as the insult because it is released from damaged cells and induces neuronal death [27]–[29], [31], [37], and inflammatory responses in microglia and macrophages [33]–[36]. Within 3 h, ATP (100 nmol in 2 µl PBS) locally induced loss of ionized calcium binding adapter molecule-1–immunopositive (Iba-1^+^) microglia as well as tyrosine hydroxylase–immunopositive (TH^+^) dopaminergic neurons (Figure 1A, asterisks: injection sites). PBS and 10 nmol ATP had little effect, while 1000 nmol ATP induced a severe reduction in TH^+^ neurons and Iba-1^+^ microglia (Figure 1A, and Figure S1). The damage area of Iba-1^+^ cells was similar to that of TH^+^ cells (Figure 1A), which was further confirmed with staining of serial sections with TH (Figure 1B left panel), Iba-1 (Figure 1B middle panel), and a combination of TH/Iba-1 (Figure 1B, right panel). Damage areas were marked with dotted lines (Fig. 1B). Electron micrographs confirmed the death of neurons and other glial cells, rather than a mere decrease in immunoreactivity of TH and Iba-1: in intact rat brain, neuron (N), astrocyte (A), and microglia (M) were shown whereas in ATP-injected brain, cellular structures were disrupted (Figure 1C). Based on these results, we employed 100 nmol ATP to induce microglia/neuron damage, and defined the area where both TH^+^ and Iba-1^+^ cells died in response to ATP injection as the core region.

**Figure 2:**
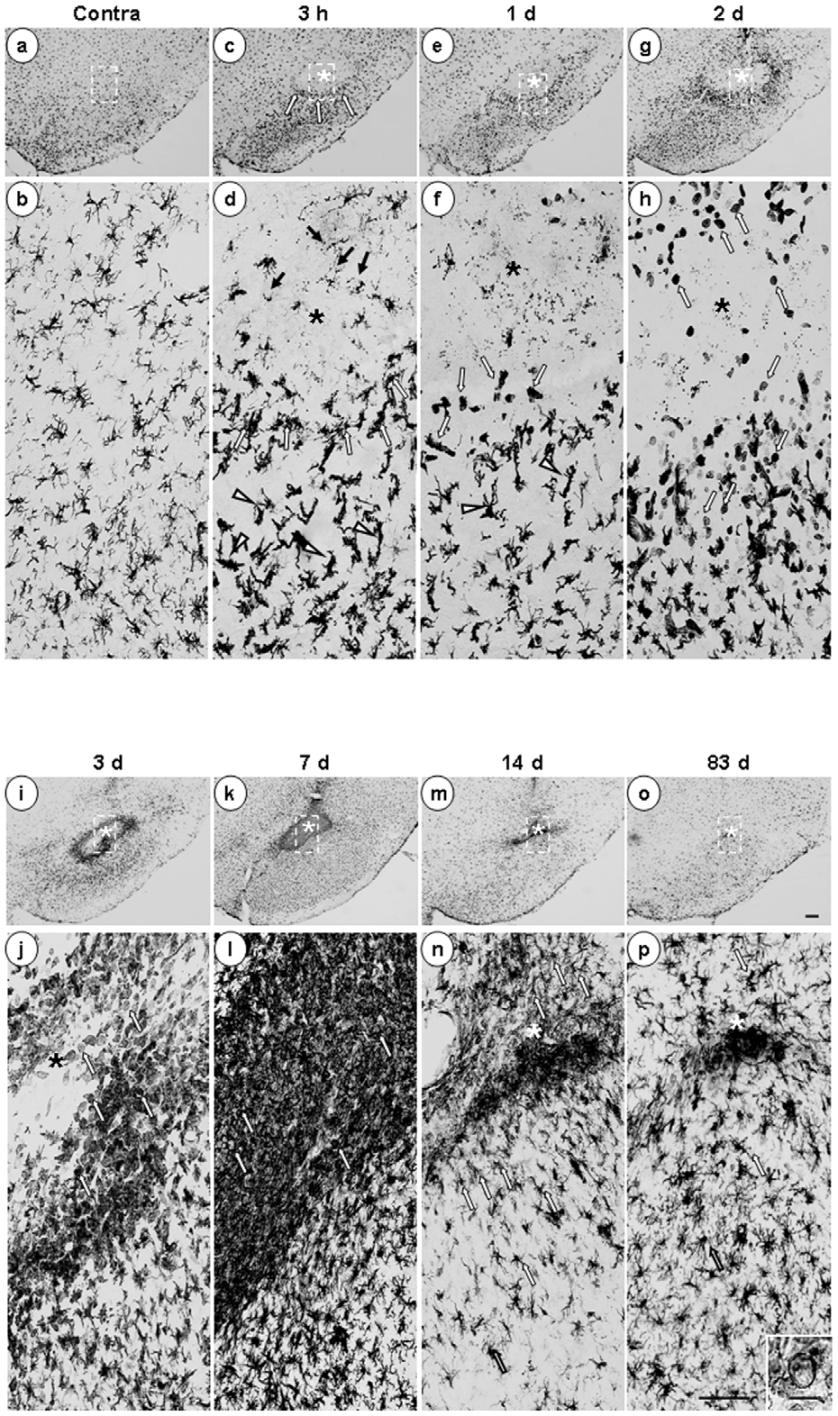
Time-dependent behavior of Iba-1^+^ cells in ATP-injected SNpc. Serial sections obtained 3 h ∼83 d after ATP injection (*, injection sites unless indicated) were processed for Iba-1 immunostaining, as described for Figure 1. Photographs of the most damaged sections were obtained. The lower panel represents higher magnification of the indicated area in the upper panel. ATP rapidly altered the behavior of Iba-1^+^ cells, and its effects lasted at least for 83 d. Black arrows in (d) show debris remained. Arrowheads in (d) and (f) indicate thick and short process-bearing Iba-1^+^ cells. White arrows represent Iba-1^+^ cells arranged around the injury site (c, d), rod-shaped cells (f), round cells (h, j, and l), and ramified cells (n, p). Inset in (p) shows that some Iba-1^+^ cells located at the needle tip still displayed a round shape. Data are representative of results obtained from at least 5 animals, unless indicated. Scale bars, 200 µm (upper panel); 100 µm (lower panel); 20 µm (inset).

Next, we investigated the time-dependent behavior of Iba-1^+^ cells from 3 h to 83 d after ATP injection. In the contralateral intact brain, Iba-1^+^ cells were evenly distributed (Figure 2a) and displayed highly ramified morphology (Figure 2b, higher magnification image of the dotted line in Figure 2a). At 3 h, in the core region (marked with asterisks), most Iba-1^+^ cells disappeared (Figure 2c, d), although the debris remained (black arrows in Figure 2d). However, in the penumbra region, processes of Iba-1^+^ cells became thick and short (arrowheads in Figure 2d), and cells near the core appeared to be arranged so that the core was isolated (white arrows in Figure 2c, d). At 1 d, some Iba-1^+^ cells near the core region became rod-shaped (white arrows in Figure 2f), and Iba-1^+^ cells beneath this area had thick, long process that headed towards the core region (arrowheads in Figure 2f). At 2 d, the number of Iba-1^+^ cells increased (Figure 2g, h), and round Iba-1^+^ cells appeared in the core and penumbra regions (white arrows in Figure 2h). At 3 d, the densities of round Iba-1^+^ cells increased (Figure 2i, j), and at 7 d, the core was densely filled with round Iba-1^+^ cells (Figure 2k, l). At 14 d, the number of round Iba-1^+^ cells was significantly reduced (Figure 2m, n), and most became ramified (white arrows in Figure 2n). At 83 d, most Iba-1^+^ cells were ramified (Figure 2p); only those Iba-1^+^ cells located at the needle tip still displayed a round shape (Figure 2o, p, and inset in Figure 2p). In the control SNpc injected with PBS (2 µl), Iba-1^+^ cells observed at 3 h had not died but their processes had retracted. At 1 d and 3 d, Iba-1^+^ cells in the PBS-injected brain once again became ramified, but had thicker processes than those in the intact (uninjected) brain (Figure S2). However, the round Iba-1^+^ cells observed in ATP-injected brains were not detectable in PBS-injected brains (Figure S2). These results indicate that Iba-1^+^ cells are vulnerable to ATP-induced damage and dramatically alter their morphology and number in response to damage.

### Monocytes but not neutrophils infiltrate the ATP-injected brain

**Figure 3:**
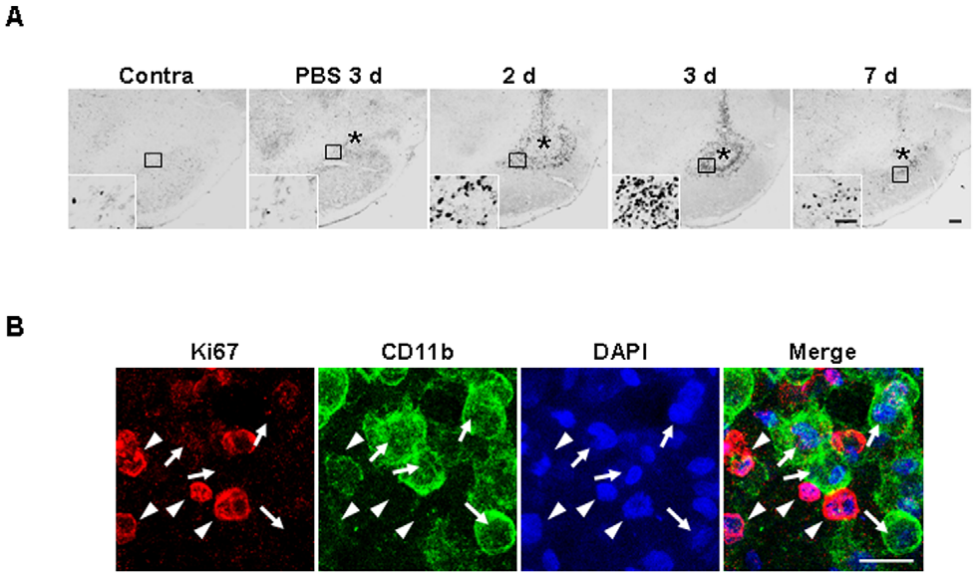
Ki-67^+^ immunoreactivity is detectable in ATP-injected SNpc, but not colocalized in CD11b^+^ cells. (**A**) Brain sections were prepared at the indicated times after ATP or PBS injection and stained with Ki-67 antibody. Photographs of the most damaged sections were obtained. The insets represent higher magnification of the area indicated in the upper panel. The number of Ki-67^+^ cells increased at 2 and 3 d, but decreased at 7 d. (**B**) Sections obtained at 3 d were double-labeled with Ki-67 and CD11b antibodies, and visualized with Alexa Fluor555- (arrowheads)- and Alexa Fluor488 (arrows)- conjugated secondary antibodies, respectively. Nuclei were labeled with DAPI. The majority of Ki-67 immunoreactivity was not detectable in CD11b^+^ cells. Scale bars, 200 µm (A); 50 µm (A, insets); and 20 µm (B).

Since the number of round Iba-1^+^ cells was significantly increased from 2 d after ATP injection, we investigated the cellular sources. Initially, we examined the possibility of proliferation of Iba-1^+^ microglia from the penumbra regions. Cells expressing Ki-67 (a cell cycle protein used as a proliferation marker) were detectable at 2 d after ATP injection, and significantly increased at 3 d, but decreased at 7 d (Figure 3A). Ki-67^+^ cells were barely detectable in PBS-injected SNpc and intact SNpc (Figure 3A). To ascertain whether microglia were Ki-67^+^, we performed double immunolabeling using CD11b and Ki-67 antibodies. Since both Iba-1 and Ki-67 antibodies were generated in the same species of animals (rabbits), CD11b antibodies generated in mice were employed to detect microglia. Interestingly, Ki-67 immunoreactivity (arrowheads in Figure 3B) did not match that of CD11b (arrows in Figure 3B). These results clearly indicate that microglia do not actively proliferate in the ATP-injected brain.

**Figure 4:**
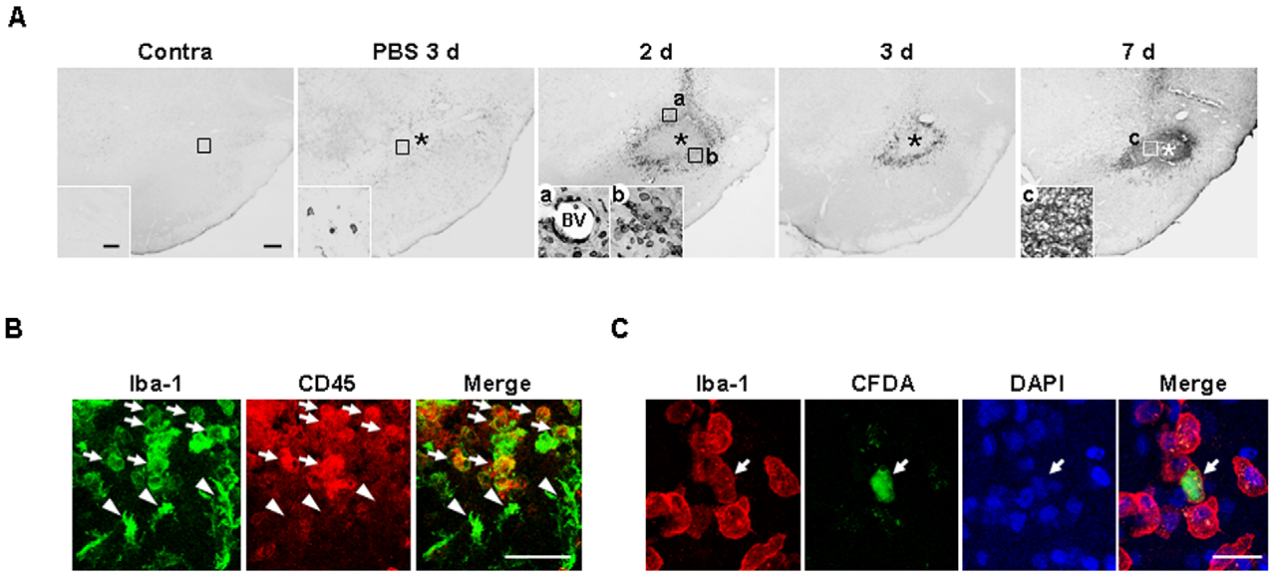
Infiltration of monocytes in ATP-injected SNpc. (**A**) Sections were obtained at the indicated times after ATP or PBS injection, and stained with CD45 antibody. Insets show higher magnification of boxed areas. (**B**) Sections were obtained 2 d after ATP injection, and double-labeled with Iba-1 and CD45 antibodies. Round Iba-1^+^ cells were CD45^+^ (arrows) whereas ramified Iba-1^+^ cells were CD45^-^ (arrowheads). (**C**) Infiltration of blood-borne monocytes in ATP-injected SNpc. CFDA-labeled monocytes were transplantated into the tail vein of rats 12 h after ATP injection as described in “Materials and Methods (Monocytes isolation and transplantation)”. Brain sections were obtained at 2 d after ATP injection, and stained with Iba-1 antibody and visualized with Alexa Fluor555- conjugated secondary antibody. Scale bars, 200 µm (A, upper panel); 50 µm (A, lower panel); 50 µm (B); 20 µm (C).

Next, we examined the possible infiltration of blood monocytes, in view of previous reports that monocytes express Iba-1 and infiltrate the damaged brain [11], [15], [17], [20], [21]. We stained sections obtained at 2–7 d after ATP injection with CD45 antibodies, since expression levels of CD45 were low in microglia and high in blood monocytes [40]–[42]. Interestingly, round CD45^+^ cells were densely located in the penumbra region at 2 d (Figure 4Aa, b) and 3 d (Figure 4A), and filled the core by 7 d (Figure 4Ac). CD45^+^ cells were tightly bound to blood vessels (Figure 4Aa) and their location at 2 d and 3 d was similar to that of round Iba-1^+^ cells, as shown in Figure 2. In the intact contralateral side, CD45^+^ cells were not detectable (Figure 4A). In PBS-injected SNpc, at 3 d, CD45^+^ cells were scattered at low density (Figure 4A). We further confirmed that round Iba-1^+^ cells detected in the penumbra region 2 d after ATP injection were co-labeled with CD45 antibody (arrows in Figure 4B), whereas Iba-1^+^ ramified cells were barely labeled (arrowheads in Figure 4B). We also confirmed monocyte infiltration in rats transplanted with CFDA (carboxyfluorescein diacetate)-labeled monocytes into the tail vein. Iba-1^+^/CFDA^+^ round cells were detectable at 2 d after ATP injection when CFDA -labeled monocytes were transplanted at 12 h after ATP injection (Figure 4C). These results indicate that Iba-1^+^/CD45^+^ monocytes actively infiltrate the ATP-injected brain.

**Figure 5:**
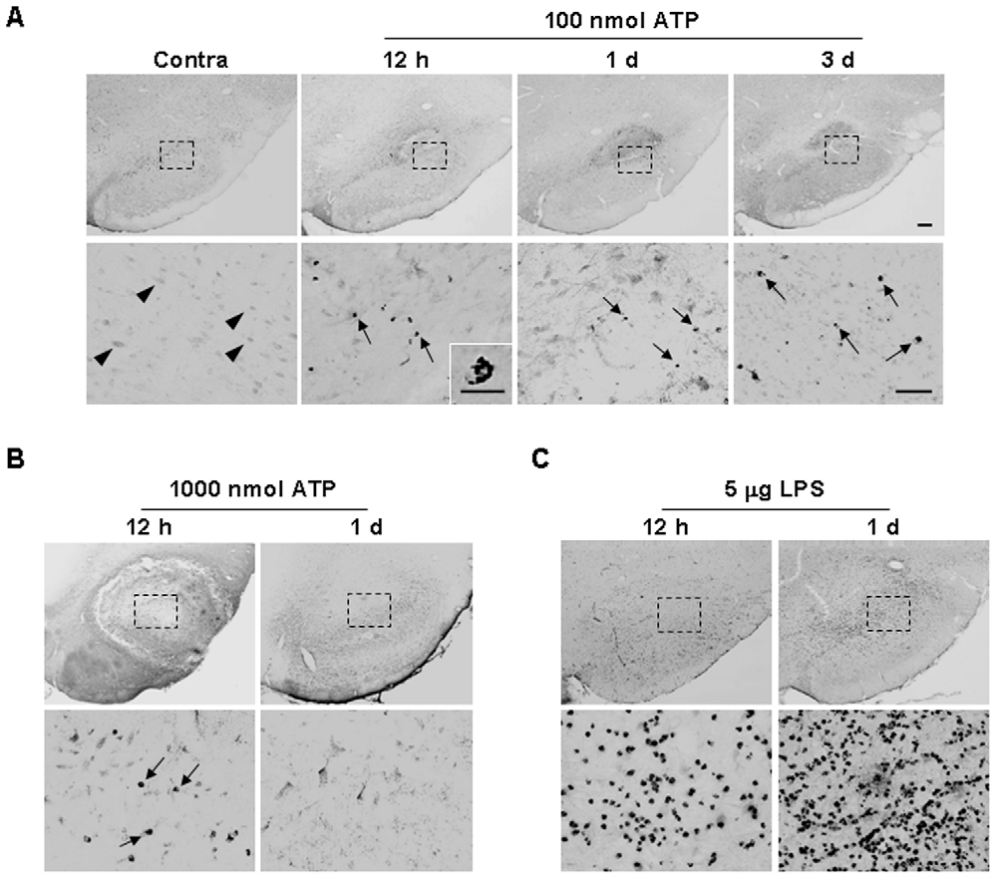
MPO^+^ neutrophils are barely detectable in ATP-injected SNpc. Brain sections were obtained at the indicated times after injection of 100 nmol ATP (**A**), 1000 nmol ATP (**B**) or 5 µg LPS (**C**) into the SNpc. Neutrophils were identified using MPO antibody (arrows). Photographs of the most damaged sections were obtained. In contrast to LPS, neutrophils were barely recruited upon treatment with ATP (100 and 1000 nmol). Lower panel is higher magnification of the upper panel. Scale bars, 200 µm (A, B, C upper panels); 50 µm (A, B, C lower panels); 10 µm (inset).

Neutrophils are initially recruited to injury sites in systemic inflammation, and LPS-injected, ischemic and traumatic brains [9]-[19], [43]. Accordingly, we examined whether neutrophils infiltrate ATP-injected SNpc. However, ATP injection did not significantly induce neutrophil recruitment. Only a few myeloperoxidase-immunopositive (MPO^+^) neutrophils were detectable in 100 nmol ATP-injected SNpc from 12 h to 3 d (arrows in Figure 5A). Even at 1000 nmol ATP, not many neutrophils were detectable (arrows in Figure 5B), in contrast to LPS-injected SNpc (Figure 5C). Neurons on the contralateral side were faintly stained with MPO antibodies (arrowheads in Figure 5A). Accordingly, neutrophil infiltration appeared variable, depending on the damage insult. These results collectively suggest that monocytes but not neutrophils infiltrate from the blood and largely contribute to the increase in Iba-1^+^ cells in the ATP-injected brain.

### Expression profiles of inflammatory mediators in ATP-injected SNpc

**Figure 6:**
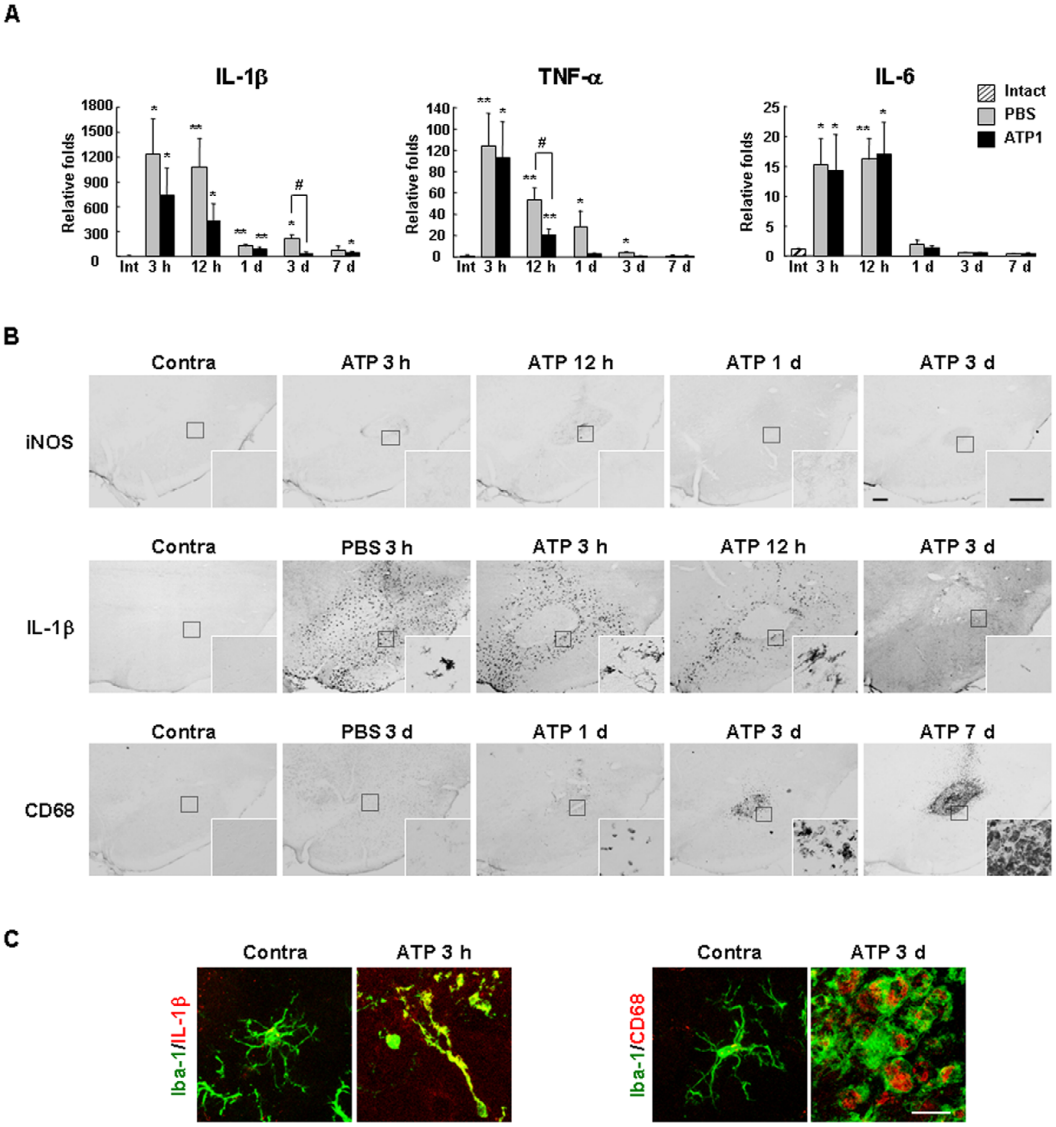
Expression of inflammatory mediators in ATP-injected SNpc. (**A**) At the indicated times after ATP or PBS injection, tissue blocks (2×2×2 mm^3^) were obtained, and mRNA levels of IL-1β, TNF-α, and IL-6 were analyzed with real time PCR. *, p<0.05 vs. intact; **, p<0.01 vs. intact; #, <0.05 between PBS vs. ATP. (**B, C**) Sections obtained at the indicated times were stained with IL-1β, iNOS or CD68 antibodies, and visualized with biotin-conjugated secondary antibodies (B) or double-labeled with combinations of IL-1β/Iba-1 or CD68/Iba-1 antibodies, and visualized with Alexa Fluor555- and Alexa Fluor488- conjugated secondary antibodies (C). Insets in (B) represent higher magnification of the boxed areas. Scale bars, 200 µm (B); 20 µm (B, insets); 20 µm (C).

Next, we investigated the mRNA expression of inflammatory mediators in the ATP-injected brain. IL-1β, TNF-α, and IL-6 mRNA reached peak levels within 3 h, were maintained (IL-1β and IL-6) or slightly decreased (TNF-α) at 12 h, and then decreased to or approached basal levels thereafter (Figure 6A). Interestingly, the expression levels of all of these cytokines were also increased in the PBS-injected brain, particularly IL-1β and TNF-α, which were higher than in ATP-injected brain at 3 d and 12 h, respectively (Figure 6A).

We further examined the expression of inflammatory mediators using an immunohistochemical approach. Interestingly, inducible nitric oxide (iNOS) immunoreactivity was not detectable in the ATP-injected SNpc for up to 7 d (Figure 6B, insets: higher magnification of boxed areas). The absence of iNOS immunoreactivity in the ATP-injected brain was not due to experimental error since iNOS was readily detected in brain sections obtained from positive control rats systemically injected with LPS (data not shown). In contrast to iNOS, IL-1β was strongly detected at 3 h, decreased at 12 h, and disappeared at 3 d (Figure 6B). IL-1β was detected in the penumbra region of the ATP-injected SNpc where microglia were morphologically activated, but not in the core where microglia had died (Figure 6B). IL-1β was also detectable in the PBS-injected SNpc, but here it was present in both the penumbra and core regions (Figure 6B). We next examined phagocytic activity by probing for CD68, a widely used marker of phagocytic activity [44]–[46]. In the ATP-injected brain, CD68 immunoreactivity was not detectable for up to 12 h, but appeared at 1 d, and significantly increased at 3–7 d (Figure 6B) in areas that were densely populated with round Iba-1^+^ cells and/or CD45^+^ cells (Figure 2 and Figure 4A). In contrast, CD68 expression was barely detectable in the PBS-injected brain (Figure 6B). In double-labeling experiments, cellular types that expressed IL-1β and CD68 were identified. As expected, IL-1β and CD68 were detected in Iba-1^+^ cells that were considered microglia and monocytes, respectively (Figure 6C).

### Delayed neuronal death is barely detectable in the penumbra region where microglia are activated and monocytes infiltrate

**Figure 7:**
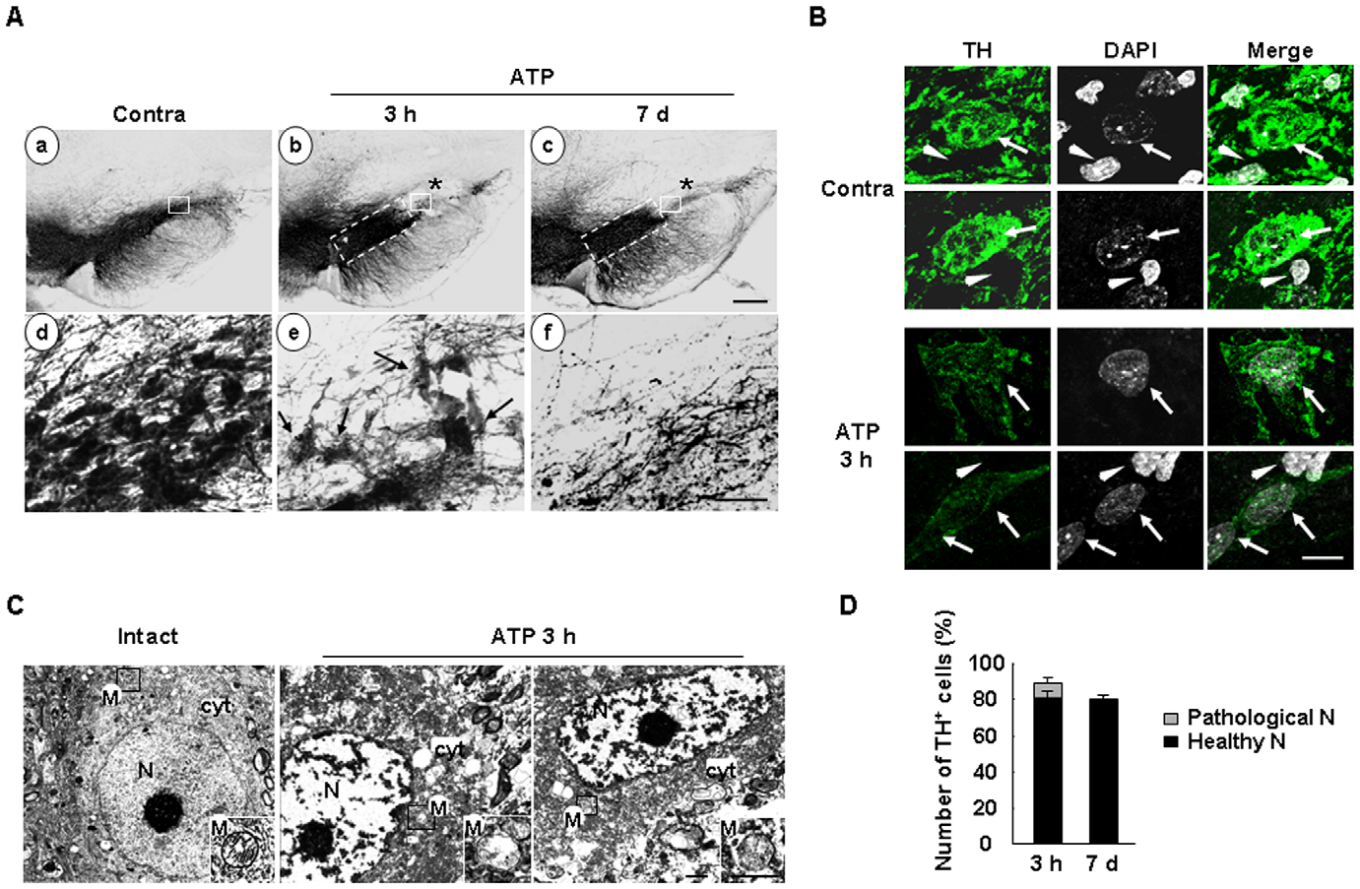
Delayed neuronal death is barely detectable in ATP-injected SNpc. (**A**) Midbrain sections including the entire SNpc were prepared at 3 h and 7 d after ATP injection, and stained with TH antibody. The contralateral side (contra) was used as the control. The lower panel represents higher magnification of boxed regions in the upper panel. The ATP-induced damage area detected at 3 h did not increase at 7 d (dotted lines in the upper panel), although TH^+^ neurons that appeared rough and unhealthy (pathological neurons, arrows in the ‘e’) disappeared at 7 d (f). (**B**) Sections obtained at 3 h were labeled with TH antibody, and the nuclei were visualized with DAPI. Nuclei of TH^+^ neurons and uncharacterized cells were indicated by arrows and arrowheads, respectively. (**C**) Electron microscopic analysis was performed in the boxed regions in intact and ATP-injected brain represented in (Aa and b). Mitochondria (M, insets) and cytosol (cyt) were indicated. (**D**) Using stereology, the total numbers of TH^+^ neurons were estimated in the contralateral and ATP-injected ipsilateral SNpc at 3 h (n = 5) and 7 d (n = 5), as described in “Materials and Methods”. The number of TH^+^ neurons in the ipsilateral side was normalized to that in the contralateral side (contra) in each animal. Pathological TH^+^ neurons detected at 3 h were counted separately. Scale bars, 200 µm (A, upper panel); 50 µm (A, lower panel); 10 µm (B); 1.7 µm (C); 0.8 µm (C, inset).

Since ATP injection did not significantly induced expression of proinflammatory mediators, such as iNOS, IL-1β, and TNF-α, we examined whether delayed neuronal damage occurred in ATP-injected brain. We compared the neuron-damaged areas at 3 h and 7 d. Interestingly, TH^+^ neuron-negative areas detected at 3 h were not increased at 7 d (Figure 7Ab, c). TH^+^ cells in the penumbra region were intact at 7 d as well as 3 h (dotted line in Figure 7Ab, c). A number of TH^+^ neuronal cell bodies detected in the core at 3 h looked unhealthy (arrows in Figure 7Ae), and disappeared at 7 d (Figure 7Af). We analyzed morphology of the nuclei of TH^+^ neurons using DAPI staining. The intensities of TH in the ipsi-lateral side slightly reduced compared to that in the contra-lateral side. On the contrary, the intensities of DAPI of TH^+^ cells in the ipsi-lateral side slightly increased compared to that in the contra-lateral side (arrows in Fig. 7B) while the intensities of non-TH^+^ cells little changed (arrowheads in Fig. 7B). We further performed the electron microscopy to verify the state of these TH^+^ neuronal cells. Electron microscopy confirmed that neurons in core region at 3 h were not healthy, since cytosolic (cyt) and mitochondrial structures (M, and insets in Figure 7C) disintegrated, compared to those in intact neurons (Figure 7C), which constitute a marker of dying cells [47]–[49]. Importantly, the characteristics of neuronal nuclei (N), pale and homogeneously dispersed karyoplasms [50], were lost (Figure 7C), which is in an accordance of the increase in DAPI intensity in ATP-injected brain shown in Figure 7B. Based on these data, we defined the unhealthy cells in core region at 3 h as ‘pathological neurons’ (arrows in Figure 7Ae). In stereological counts, only the pathological neurons detected at 3 h disappeared at 7d, and the total numbers of healthy TH^+^ neurons estimated at 3 h and 7 d were similar, specifically, 81.1±2.8% (n = 5) and 80±1.9% (n = 5) of that in the contralateral side at 3 h and 7 d, respectively (Figure 7C). Thus, we concluded that TH^+^ neurons in the SN remained in the core at 3 h after ATP injection were dead and/or dying (pathological) neurons.

**Figure 8:**
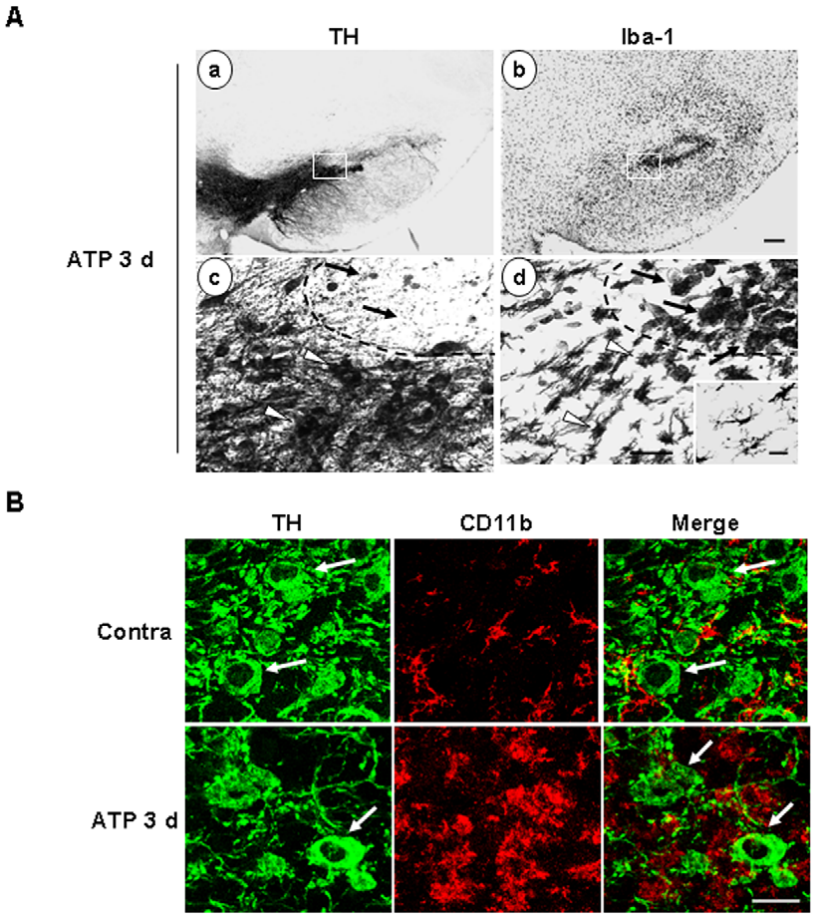
TH^+^ dopaminergic neurons are healthy in the area where Iba-1^+^ cells are morphologically activated within ATP-injected SNpc. Brain sections were prepared 3 d after ATP injection. (**A**) Serial sections were stained with TH (a, c) or Iba-1 (b, d) antibodies, and visualized with biotin-conjugated secondary antibodies. Lower panels represent higher magnification of the boxed areas in the upper panels. In the area specified with arrows, TH^+^ neurons disappeared (c) and round Iba-1^+^ cells were densely located (d). In the area indicated by white arrowheads, TH^+^ neurons were healthy (c) and Iba-1^+^ cells were morphologically activated (d). The inset in (d) presents the morphology of Iba-1^+^ cells in contralateral SNpc. (**B**) Sections were double-labeled with TH and CD11b antibodies. Photographs were obtained at the area indicated with arrowheads in the ipsilateral side in (A) and contralateral side (contra). TH^+^ neuronal cell bodies are indicated with arrows. Scale bars, 200 µm (A, upper panel); 50 µm (A, lower panel), 20 µm (A, inset), 50 µm (B).

Next, we examined whether microglial activation/monocyte infiltration occurs in the penumbra region containing live TH^+^ neurons. Serial sections were obtained from ATP-injected SNpc at 3 d, and stained with TH and Iba-1 antibodies. Lower panels (Figure 8Ac, d) are higher magnification images of the indicated areas in the upper panels (Figure 8Aa, b). At 3 d, TH^+^-neuron negative and positive areas were clearly detected (dotted lines in Figure 8Ac). In TH^+^ neuron-negative area, round Iba-1^+^ cells reappeared (arrows in Figure 8Ad) while in TH^+^ neuron-positive area, morphologically activated thick process-bearing Iba-1^+^ cells were detected (arrowheads in Figure 8Ad). The morphology of resting microglia in the contralateral side was shown in the inset (Figure 8Ad). Double immunostaining experiments disclosed intact TH^+^ neuronal cell bodies (arrows in Figure 8B) with co-existence of activated CD11b^+^ cells (Figure 8B), which indicate that morphologically activated Iba-1^+^ cells were not cytotoxic to the surrounding neurons.

**Figure 9:**
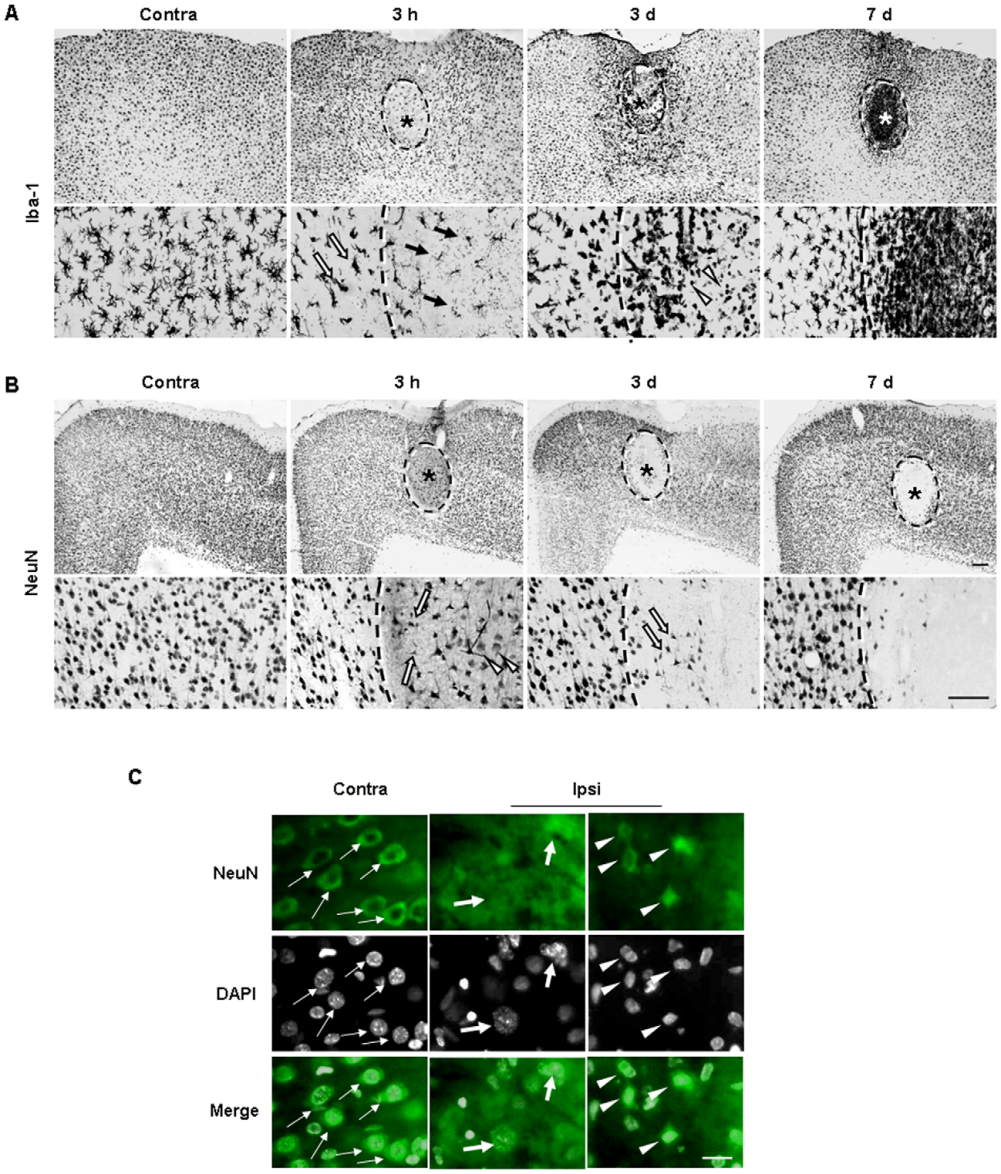
Effect of ATP on behavior of neurons and Iba-1^+^ cells in the cortex. ATP was injected into the cortex, and serial sections prepared at the indicated times, and stained with Iba-1 (A) and NeuN (B) antibodies. The contralateral side (contra) was used as a control. (**A**) Within 3 h, Iba-1^+^ cells underwent death in the core (black arrows) and activated in the penumbra region (white arrows). At 3 d, round Iba-1^+^ cells were detectable in the core (arrowheads), and Iba-1^+^ cells filled the core at 7 d. (**B**) Neuron damaged areas were marked with dotted lines. At 3 h and 3 d, swollen (white arrowheads) and shrunken (white arrows) neuronal cell bodies were detectable in the core. At 3 d and 7 d, healthy neurons were observed in the penumbra region (outside of dotted lines). (**C**) Sections obtained at 3 h were labeled with NeuN antibody, and the nuclei were detected with DAPI. The nuclei of NeuN^+^ cells in the contralateral side were round and the boundary was clear (thin arrows). However, the nuclei of NeuN^+^ cells in the contralateral side were either swollen (thick arrows) or shrunk (arrowheads). Scale bars, 200 µm (A and B, upper panel); 100 µm (A and B, lower panel); 20 µm (C).

We further examined ATP-induced inflammatory responses and neuronal damage in the cortex. The behavior of NeuN^+^ neurons and Iba-1^+^ cells was similar to that in the SNpc. In the core region, Iba-1^+^ cells died within 3 h after ATP injection (black arrows in Figure 9A). Round Iba-1^+^ cells were detected at 3 d in the core region (white arrowheads in Figure 9A). In the penumbra region, processes of Iba-1^+^ cells became thick and short within 3 h (white arrows in Figure 9A), and further decreased at 3 d (Figure 9A). However, at 7 d, Iba-1^+^ cells became ramified again (Figure 9A). Although NeuN^+^ cells were detected in the injection core at 3 h after ATP injection, these cells were either swollen (arrowheads in the lower panels in Figure 9B) or shrunken (arrows in the lower panels in Figure 10A), compared to NeuN^+^ cells in the contralateral side (Figure 9B). We further analyzed morphological changes of the nuclei using NeuN/DAPI staining. In the contralateral side, nuclei of NeuN^+^ cells were round and healthy (thin arrows in Figure 9C). However, in the core of the ipsilateral side, nuclei of NeuN^+^ cells were either swollen (thick arrows in Figure 9C) or shrunk (arrow heads in Figure 9C), a sign of necrosis and apoptosis, respectively [51], [52]. Therefore, these results suggest that remaining cells detected in the core at 3 h already underwent dying processes due to direct toxicity of ATP. Importantly, the NeuN-negative area detected at 3 d and 7 d did not increase, compared to the damaged area at 3 h (dotted lines in Figure 9B). At 3 d, the NeuN^+^ cells in the penumbra region were healthy (Figure 9B), while in the core region, swollen NeuN^+^ cell bodies disappeared and some shrunken cell bodies were remained (white arrows in Figure 9B). At 7 d, NeuN^+^ cells in the penumbra region were still healthy, while in the core region, most NeuN^+^ shrunken cells disappeared (Figure 9B). These results indicate that ATP induces brain injury and behavioral changes of microglia and monocytes but that activated microglia and infiltrated monocytes do not generate delayed neuronal damage in either SNpc or the cortex.

## Discussion

In this study, we examined the behavior of neurons, microglia and other inflammatory cells in the ATP-injected brain as a model system that mimics the brain injury. The major findings are as follows: (1) microglia as well as neurons underwent death in the damaged core; (2) monocytes as well as microglia contributed to brain inflammation; and (3) inflammatory responses of microglia and/or monocytes were not cytotoxic since there was no sign of delayed neuronal death in the penumbra region.

In ATP-injected SNpc, microglia acutely died as early as neurons, and round Iba-1^+^ cells filled the microglia-dead area at 3–7 d (Figure 1, 2). Although the number of Ki67^+^ cells increased in 2 d and 3 d after ATP injection (Figure 3A), Ki67 immunoreactivity was not detected in CD11b^+^ cell (Figure 3B), which indicated that resident microglia did not actively proliferate in ATP-injected brain. In accordance with a recent report that astrocytes and oligodendrocytes actively proliferate in injured brain [53], we also detected Ki67 immunoreactivity in GFAP^+^ astrocytes in ATP-injected brain (data not shown). The round Iba-1^+^ cells filled the microglia-dead area were identified as infiltrated monocytes in several ways. Iba-1^+^ round cells detected at 3 d expressed high levels of CD45 (Figure 4). CD45 was used as a marker of monocytes, in view of the earlier finding that resident microglia display low CD45 expression [40]–[42]. In addition, CFDA-labeled monocytes transplanted into the tail vein were detected in ATP-injected brain (Figure 4). We also could exclude the possibility that ATP could enhance CD45 expression level in microglia since CD45 expression was significantly stronger in monocytes and microglia did not enhance CD45 expression even in the presence of ATP (Figure S3).

The next arising issue was to understand the roles of resident microglia activated in the penumbra region and infiltrated monocytes in the core region. Inflammatory mediators from microglia, such as nitric oxide and prostaglandins produced by iNOS and COX-2, respectively, are cytotoxic in various animal models [3], [5], [6], [7]. Therefore, brain inflammation is suggested as a cause of delayed neuronal injury that follows primary injury. However, this general concept is not always true. In animal models of Alzheimer's disease, amyotrophic lateral sclerosis (ALS), and multiple sclerosis, microglia possibly function in the maintenance, repair or regeneration of the microenvironment [21], [54], [55]. As observed in this study, the injury site and/or loss of neurons in ATP-injected SNpc and cortex did not increase between 3 h and 7 d (Figure 7 and 9). In view of these inconsistent findings, it is important to consider the factors that determine the presence or absence of delayed injury. Firstly, differences in the extent of neutrophil infiltration may influence the incidence of delayed injury since neutrophils release neurotoxic factors, such as reactive oxygen species, proteolytic enzymes, iNOS, and other proinflammatory mediators [9]–[19]. Previously, we have reported that LPS slowly (at around 12–24 h) induced dopaminergic neuronal death in the SN, but did not induce neuronal death in the cortex [18]. LPS induced tremendous neutrophil infiltration in the SN but much less in the cortex, and blocking of neutrophil infiltration reduced neuronal death in the SN [18]. In ischemic, traumatic, quinolic acid- and LPS-injected brains, neutrophils infiltrate and induce delayed neuronal damage [13], [14], [17], [18]. Furthermore, neutrophil depletion reduces brain injury in these conditions [12], [13], [18], [56]. Compared to LPS, ATP is a weak inflammatory stimulator (Figure S4), and barely recruited neutrophils (Figure 5). Thus, the absence of neutrophil infiltration may not induce delayed neuronal death in ATP-injected brain. Secondly, the extent of damage could determine the absence or presence of delayed neuronal death. When the amount of ATP increased 10 folds, from 50 mM to 500 mM, absolute neuron-damage areas increased (Figure S5). However, neuron-damage area detected at 7 d rather slightly reduced compared to that at 3 h (Figure S5). Based on these results, we concluded that the extent of damage may not determine the absence or presence of delayed neuronal death. Another factor that determines the presence or absence of delayed neuronal damage could be the balance between neurotoxic and neuroprotective factors produced by inflammatory cells. ATP injection barely induced cytotoxic iNOS expression (Figure 6), possibly because ATP is a weak inflammatory stimulus for induction of iNOS expression (Figure S4). Furthermore, astrocytes have been reported to suppress expression of iNOS [57], [58]. Although ATP induced mRNA and/or protein expression of IL-1β, TNF-α, and IL-6 (Figure 6), PBS, which did not induce neuronal death, also induced these cytokines, and to a greater degree than ATP. These findings suggest that IL-1β, TNF-α, and IL-6 induction mechanisms are sensitive enough that the expression of these cytokines can be stimulated by mechanical damage (Figure 6A, B). Furthermore, these cytokines are rather neuroprotective than neurotoxic since neurons in the penumbra region where these cytokines were expressed were healthy (Figure 6, 7, 8) and PBS did not induce neuronal damage (Figure S1). These findings are in accordance with previous reports showing that TNF-α and IL-6 released from ATP-treated microglia could be neuroprotective [35], [59], [60]. In particular, ATP-stimulated microglia have been reported to protect neurons from glutamate-induced excitotoxicity [60], consistent with the idea that growth factors produced by activated microglia could exert beneficial effects on neurons in injury states.

Monocytes may act in repair by scavenging injured cells and cell debris with strong phagocytic activity, as in systemic inflammation where monocytes contribute to resolution and repair by removing apoptotic cells and facilitating angiogenesis [61], [62]. In the ATP-injected brain, monocytes expressed CD68, a marker of phagocytic activity [44]–[46]. (Figure 6). Accordingly, pathologically shrunken neurons disappeared at around 3–7 d when monocytes infiltrated in the SNpc (Figure 4, and 7). The remaining monocytes may differentiate into microglia or undergo cell death after scavenging and/or repairing damaged brain, since the number of round cells abruptly decreased between 7–14 d and ramified cells were detected around the injection sites (Figure 2). Accordingly, at 83 d, the morphology of Iba-1^+^ cells in the most damaged area was similar to that in intact brain (Figure 2).

Although brain inflammation accompanies brain injury, different cellular components (microglia, astrocytes, monocytes, and neutrophils) and different factors (proinflammatory and neurotrophic) could participate in inflammatory processes depending on the nature of the insult or injury states. On the basis of the results presented here and in previously reported studies [17], [18], we suggest that the inflammatory responses of monocytes and microglia are not neurotoxic. These findings highlight the importance of distinguishing between inflammatory factors that are harmful and beneficial to neurons in diverse brain diseases, and developing methods to selectively regulate harmful and beneficial factors.

## Supporting Information

Figure S1 Dose-dependent death of dopaminergic neurons and microglia in the SNpc induced by ATP. ATP (10 or 1000 nmol in 2 µl PBS) or PBS (2 µl) was unilaterally injected into SNpc (*, injection sites), and brains were obtained 3 h (A) or indicated times (B) after the injection. Brain sections (30 µm thickness) of the midbrain including the entire SN were prepared, every sixth serial section selected and stained with TH and/or Iba-1 antibodies, and visualized with biotin-conjugated secondary antibodies. Photographs of the most damaged sections were obtained. The contralateral side (contra) was used as a control (B). Scale bars, 200 µm.
(1.18 MB TIF)

Figure S2 Behavior of Iba-1^+^ cells in PBS-injected SNpc. Brain sections (30 µm) were obtained at the indicated times after PBS (2 µl) injection, and stained with Iba-1 antibody. Photographs of the most damaged sections were obtained unless indicated. The lower panel represents higher magnification of the area indicated in the upper panel. *, Injection sites. Scale bars, 200 µm (upper panel); 50 µm (lower panel).
(2.07 MB TIF)

Figure S3 ATP does not change CD45 expression levels in microglia and monocytes. Primary microglia were cultured from the cerebral cortices of 1 to 3 day-old Sprague Dawley rats. Rat blood monocytes were isolated by density gradient centrifugation, as described in “Materials and Methods (Monocytes isolation and transplantation)”. CFDA (green)-labeled microglia and blood monocytes were co-cultured and treated with 100 µM ATP for 12 h or left untreated, and stained with CD45 antibody. Cells were CFDA-labeled microglia (arrowheads) displayed weak CD45 expression, even in the presence of ATP, while monocytes strongly expressed CD45 (arrows). Scale bars, 20 µm
(0.82 MB TIF)

Figure S4 ATP induces IL-1β, but not iNOS in primary cultured microglia. Primary cultured microglia were treated with 100 µM ATP (in C, 100 µM or 1 mM) or 10 ng/ml LPS. At the indicated times (A, B) or 24 h (C) after the treatment, IL-1β, TNF-α, and iNOS mRNA (A) and protein (B) expression were determined with RT-PCR and Western blot, respectively. (C) The amount of nitrite formed from nitric oxide was measured by mixing the microglia culture medium (50 µl) with an equal volume of Griess reagent (0.1% naphthylethylene diamine, 1% sulfanilamide and 2.5% H3PO4). The optical density was measured at 540 nm. Values are presented as means ± SEM of three samples. #, p<0.05 vs. values from untreated or ATP-treated microglia.
(0.31 MB TIF)

Figure S5 No correlation between extent of brain damage and delayed neuronal death. (A) Brain sections were prepared at 3 h and 7 d after ATP (50 mM or 500 mM) injection into the cortex, and stained with NeuN antibody. The contralateral side (contra) was used as the control. Absolute damage areas increased with the increase in amount of ATP (from 50 mM to 500 mM). However, NeuN-negative areas did not increase between 3 h and 7 d. (B) Every sixth cortical sections (bregma AP, +2.52 ∼−0.60 mm) were stained with Cresyl Violet for Nissl staining. We used Nissl staining instead of NeuN antibody staining since 500 mM ATP induced severe damage in large area, thus damaged tissues were sometimes lost during the NeuN antibody staining processes. Nissl-negative areas were measured at 3 h and 7 d after ATP injection using Axiovision image analysis software (version 4.7.2; Zeiss). Neuron-damage areas induced by 500 mM ATP were rather slightly reduced at 7 d compared to that at 3 h. (*, P<0.01). Scale bar, 200 µm.
(0.50 MB TIF)
