# Supplementary figures and images for: Inflammatory Responses Are Not Sufficient to Cause Delayed Neuronal Death in ATP-Induced Acute Brain Injury
Source: PLoS One. 2010 Oct 29;5(10):e13756. doi: 10.1371/journal.pone.0013756 (PMC2966428; doi:10.1371/journal.pone.0013756)

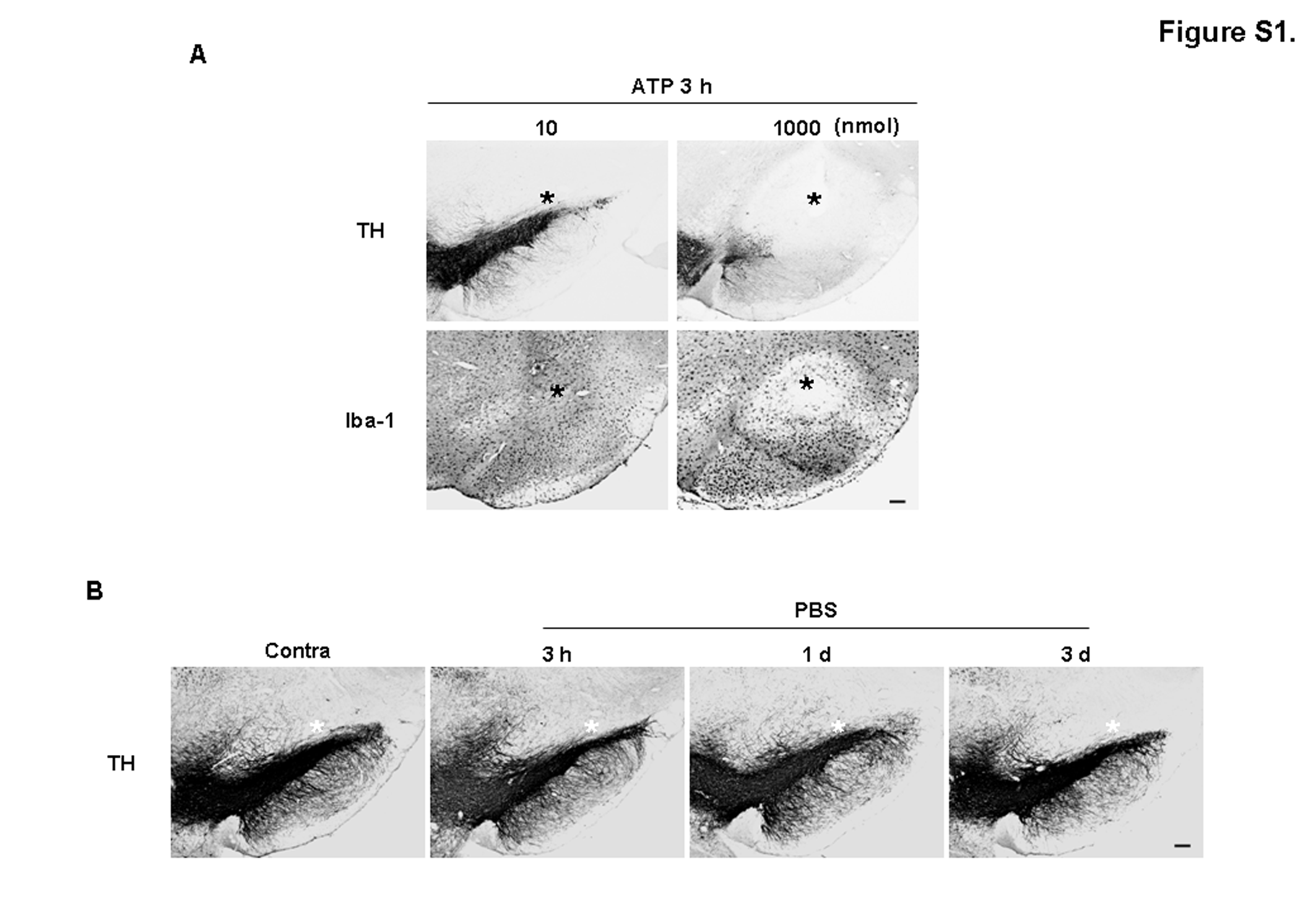

Supplement: Figure S1 — Dose-dependent death of dopaminergic neurons and microglia in the SNpc induced by ATP. ATP (10 or 1000 nmol in 2 µl PBS) or PBS (2 µl) was unilaterally injected into SNpc (*, injection sites), and brains were obtained 3 h (A) or indicated times (B) after the injection. Brain sections (30 µm thickness) of the midbrain including the entire SN were prepared, every sixth serial section selected and stained with TH and/or Iba-1 antibodies, and visualized with biotin-conjugated secondary antibodies. Photographs of the most damaged sections were obtained. The contralateral side (contra) was used as a control (B). Scale bars, 200 µm. (1.18 MB TIF) [file pone.0013756.s001.tif]

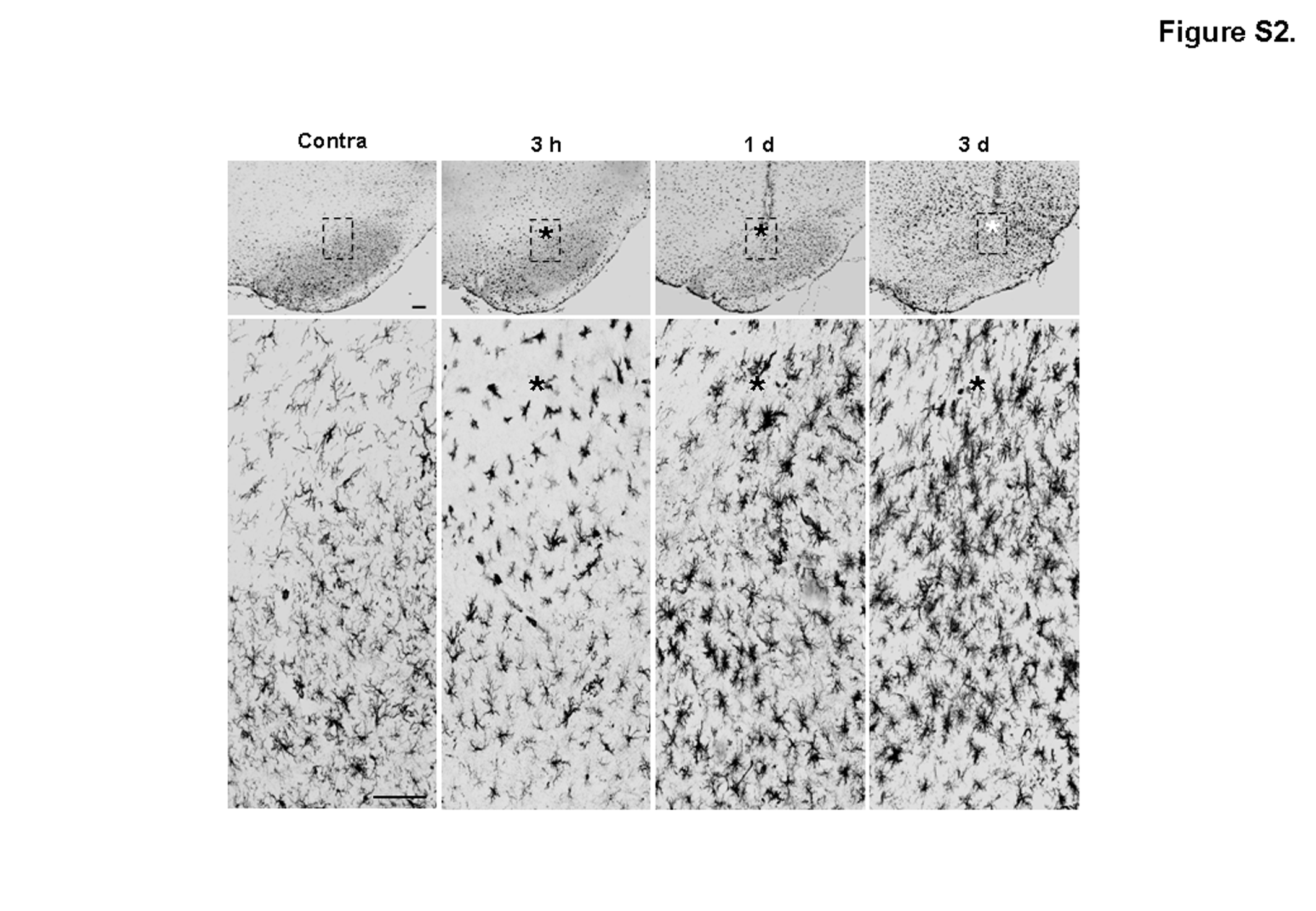

Supplement: Figure S2 — Behavior of Iba-1+ cells in PBS-injected SNpc. Brain sections (30 µm) were obtained at the indicated times after PBS (2 µl) injection, and stained with Iba-1 antibody. Photographs of the most damaged sections were obtained unless indicated. The lower panel represents higher magnification of the area indicated in the upper panel. *, Injection sites. Scale bars, 200 µm (upper panel); 50 µm (lower panel). (2.07 MB TIF) [file pone.0013756.s002.tif]

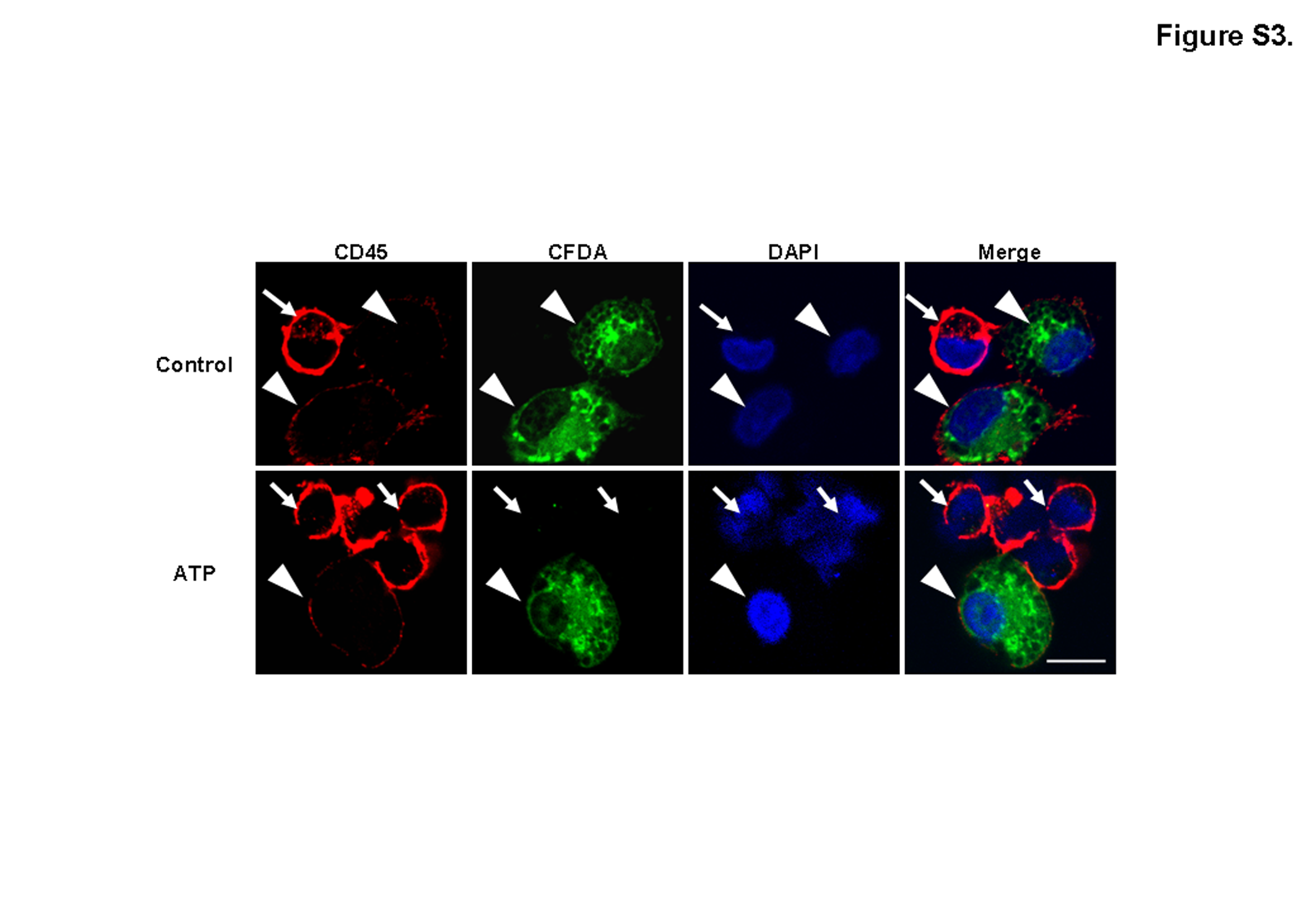

Supplement: Figure S3 — ATP does not change CD45 expression levels in microglia and monocytes. Primary microglia were cultured from the cerebral cortices of 1 to 3 day-old Sprague Dawley rats. Rat blood monocytes were isolated by density gradient centrifugation, as described in “Materials and Methods (Monocytes isolation and transplantation)”. CFDA (green)-labeled microglia and blood monocytes were co-cultured and treated with 100 µM ATP for 12 h or left untreated, and stained with CD45 antibody. Cells were CFDA-labeled microglia (arrowheads) displayed weak CD45 expression, even in the presence of ATP, while monocytes strongly expressed CD45 (arrows). Scale bars, 20 µm (0.82 MB TIF) [file pone.0013756.s003.tif]

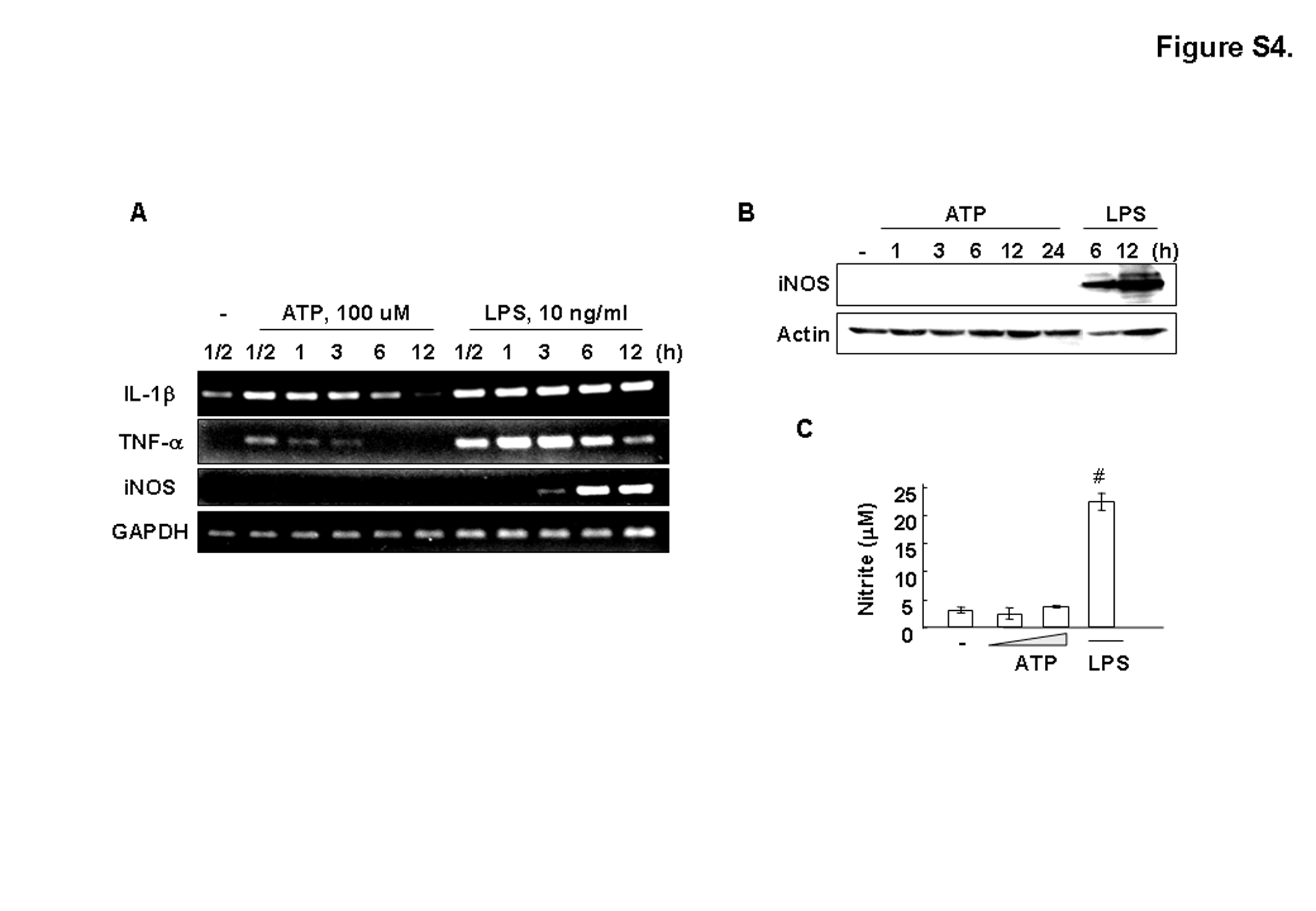

Supplement: Figure S4 — ATP induces IL-1β, but not iNOS in primary cultured microglia. Primary cultured microglia were treated with 100 µM ATP (in C, 100 µM or 1 mM) or 10 ng/ml LPS. At the indicated times (A, B) or 24 h (C) after the treatment, IL-1β, TNF-α, and iNOS mRNA (A) and protein (B) expression were determined with RT-PCR and Western blot, respectively. (C) The amount of nitrite formed from nitric oxide was measured by mixing the microglia culture medium (50 µl) with an equal volume of Griess reagent (0.1% naphthylethylene diamine, 1% sulfanilamide and 2.5% H3PO4). The optical density was measured at 540 nm. Values are presented as means ± SEM of three samples. #, p<0.05 vs. values from untreated or ATP-treated microglia. (0.31 MB TIF) [file pone.0013756.s004.tif]

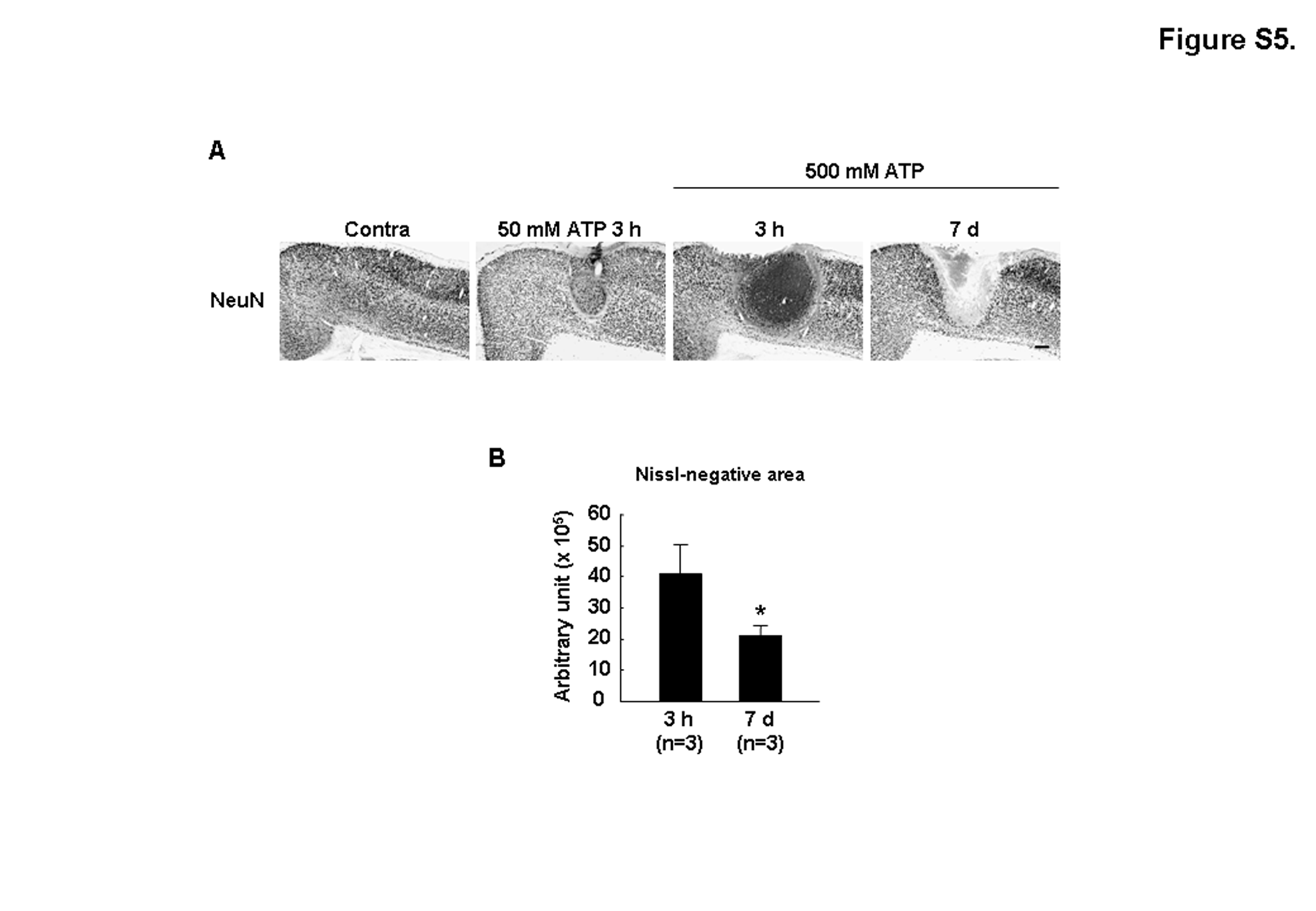

Supplement: Figure S5 — No correlation between extent of brain damage and delayed neuronal death. (A) Brain sections were prepared at 3 h and 7 d after ATP (50 mM or 500 mM) injection into the cortex, and stained with NeuN antibody. The contralateral side (contra) was used as the control. Absolute damage areas increased with the increase in amount of ATP (from 50 mM to 500 mM). However, NeuN-negative areas did not increase between 3 h and 7 d. (B) Every sixth cortical sections (bregma AP, +2.52 ∼−0.60 mm) were stained with Cresyl Violet for Nissl staining. We used Nissl staining instead of NeuN antibody staining since 500 mM ATP induced severe damage in large area, thus damaged tissues were sometimes lost during the NeuN antibody staining processes. Nissl-negative areas were measured at 3 h and 7 d after ATP injection using Axiovision image analysis software (version 4.7.2; Zeiss). Neuron-damage areas induced by 500 mM ATP were rather slightly reduced at 7 d compared to that at 3 h. (*, P<0.01). Scale bar, 200 µm. (0.50 MB TIF) [file pone.0013756.s005.tif]
